# Supplementary material for: The Effect of Spring Barley Fertilization on the Content of Polycyclic Aromatic Hydrocarbons, Microbial Counts and Enzymatic Activity in Soil
Source: Int J Environ Res Public Health. 2023 Feb 21;20(5):3796. doi: 10.3390/ijerph20053796 (PMC10001663; doi:10.3390/ijerph20053796)
Supplement: Supplementary file 1 [file ijerph-20-03796-s001.zip › ijerph-2170128-supplementary.pdf]

Table S1. Dates of agricultural operations in the production of spring barley.

Agricultural operation	Date
Fall plowing	27 October 2014
Cultivation unit, harrow	20 March 2015
NPK fertilization (according to plan), cultivation unit, harrow	21 March 2015
Sowing of spring barley cv. Olof at 350 seeds m^{-2} - 164 kg ha^{-1}	24 March 2015
Application of Mustang herbicide, 0.5 l ha^{-1}	11 May 2015
Top dressing with ammonium nitrate (34% N) (according to plan)	22 May 2015
Application of Decis Mega 50 EW pesticide, 0.1 l ha^{-1} (cereal leaf beetle)	10 June 2015
Harvest with a plot harvester	5 August 2015

Table S2. Phenological growth stages of spring barley cv. Olof.

Growth stage	Date
BBCH 10	15 April 2015
BBCH 16	20 April 2015
BBCH 20	4 May 2015
BBCH 25	9 May 2015
BBCH 30	20 May 2015
BBCH 35	23 May 2015
BBCH 51	8 June 2015
BBCH 55	12 June 2015
BBCH 61	14 June 2015
BBCH 65	17 June 2015
BBCH 69	19 June 2015
BBCH 73	11 July 2015
BBCH 75	15 July 2015
BBCH 81	19 July 2015
BBCH 85	23 July 2015

BBCH 87	27 July 2015
BBCH 89	30 July 2015

Table S3. Mean daily temperature and total precipitation in the study area in 2015.

Day	Month											
	Jan	Feb	March	April	May	June	July	Aug	Sept	Oct	Nov	Dec
Temperature (°C)												
1	1.1	-0.9	3.8	3.0	10.2	16.6	16.8	16.5	27.5	9.7	8.2	3.0
2	3.1	-1.4	4.8	2.5	6.5	16.4	18.2	19.4	18.4	11.3	6.1	2.6
3	3.0	-1.0	2.4	2.6	6.8	19.8	22.2	24.0	16.9	11.2	4.6	6.2
4	1.5	-2.2	1.2	1.7	11.8	13.4	24.7	24.4	14.2	14.6	1.5	6.2
5	-0.4	-7.4	-0.5	2.2	15.2	15.4	26.1	25.4	14.8	11.1	4.2	6.1
6	-7.8	-3.3	0.1	2.9	15.2	20.8	22.5	22.7	12.3	10.4	3.1	7.4
7	-7.3	-1.5	5.8	4.1	13.1	17.2	20.0	26.7	12.0	4.7	5.3	5.6
8	-1.2	-1.0	8.9	7.6	10.7	12.2	19.4	30.2	11.2	3.8	10.9	2.6
9	2.3	-0.4	8.3	4.8	13.9	11.8	15.8	23.1	11.4	2.1	8.0	4.4
10	5.7	1.2	7.3	9.4	13.4	13.6	12.8	20.8	13.4	0.1	11.1	3.4
11	3.8	1.0	5.4	12.0	13.0	15.7	14.5	23.0	14.2	0.7	12.6	1.1
12	2.8	0.5	3.5	9.7	14.7	18.2	15.4	23.7	13.7	0.7	10.7	3.5
13	6.6	-0.7	2.3	7.4	11.4	20.2	16.3	21.4	13.8	4.4	7.0	2.2
14	5.0	1.0	4.7	5.4	10.1	20.3	16.3	19.4	14.6	6.6	6.5	0.0
15	1.1	-1.3	6.2	9.3	7.9	15.1	16.1	22.9	17.2	6.8	5.3	-0.4
16	5.5	-1.8	6.8	7.7	8.7	12.0	16.3	23.5	17.2	7.8	4.2	-3.3
17	3.6	-2.6	7.2	5.8	10.8	14.9	18.0	21.6	22.1	8.9	8.5	2.9
18	-0.2	-0.5	5.1	3.6	10.6	15.5	20.6	19.2	17.7	8.8	9.2	8.4
19	-0.3	2.1	2.3	4.5	15.9	13.1	19.0	18.1	15.2	8.2	8.8	8.9
20	-1.1	1.6	3.7	6.2	13.2	14.3	16.5	18.4	13.5	7.1	6.5	7.7
21	0.4	3.7	4.9	5.9	11.5	14.7	16.9	16.9	11.3	6.2	3.9	5.8
22	-0.9	2.2	-1.3	6.3	13.6	15.7	19.8	16.1	11.4	7.4	2.5	7.4
23	-0.3	3.2	1.9	10.3	13.2	14.2	19.6	17.7	15.3	9.6	0.1	9.6
24	-3.3	3.3	5.2	11.1	14.0	13.7	18.6	23.0	14.2	7.4	-0.2	4.5
25	-2.9	3.1	6.2	13.6	14.4	14.7	20.8	19.7	11.8	6.9	-0.9	6.1
26	-1.3	2.9	10.9	15.2	12.6	15.8	17.3	17.5	12.0	5.6	0.0	7.2
27	-2.2	3.7	7.0	15.6	10.7	15.5	15.2	21.6	10.3	4.0	0.9	5.9
28	0.2	4.5	4.8	11.0	11.6	18.2	17.4	20.5	9.8	4.6	-1.0	3.4
29	0.4	-	4.6	7.6	14.3	15.9	17.1	17.1	9.2	4.8	2.3	-2.8
30	-0.2	-	6.7	7.4	14.5	16.7	14.3	20.9	10.3	5.1	4.4	-4.2
31	0.6	-	3.1	-	12.2	-	14.1	23.7	-	4.9	-	-3.9

**Monthly average**	0.6	0.3	4.6	7.2	12.1	15.7	18.0	21.3	14.2	6.6	5.1	3.8

Table S3 continued

Day	Month											
	**Jan**	**Feb**	**March**	**April**	**May**	**June**	**July**	**Aug**	**Sept**	**Oct**	**Nov**	**Dec**
Precipitation (mm)												
1	0.0	0.0	0.0	0.0	4.8	0.1	0.0	0.0	3.0	0.0	0.0	2.5
2	0.0	0.0	9.0	2.5	0.0	0.0	0.0	0.0	0.0	0.0	0.0	3.6
3	0.0	3.6	4.2	0.0	0.0	0.0	0.0	0.0	0.0	0.0	0.0	2.2
4	0.0	0.0	3.0	3.2	0.8	0.0	0.0	0.0	0.0	0.0	0.0	1.0
5	0.1	0.0	2.0	2.2	1.5	0.0	0.0	0.0	0.0	0.0	0.0	0.0
6	0.0	0.0	1.0	0.0	0.0	0.0	0.6	0.0	23.0	0.0	9.6	0.0
7	1.5	1.0	0.0	0.2	0.0	0.0	0.2	0.0	13.0	0.0	6.5	0.0
8	9.5	1.1	0.0	0.0	0.0	0.0	5.5	2.0	4.0	0.0	0.0	0.0
9	6.8	0.0	0.0	0.0	0.1	0.0	5.0	0.0	0.0	0.0	8.6	0.0
10	2.8	0.0	0.0	0.0	0.0	0.0	2.3	0.0	0.0	0.0	6.0	0.0
11	0.0	0.0	0.0	1.2	0.0	0.0	0.0	0.0	0.0	0.0	7.5	4.7
12	0.0	0.0	0.0	0.0	3.5	10.0	6.5	0.0	0.0	0.1	1.3	2.0
13	0.0	0.0	0.0	0.0	0.0	0.0	11.0	0.0	0.0	0.0	4.5	15.3
14	0.2	0.0	0.0	2.5	0.1	0.0	0.0	0.0	3.8	0.0	0.0	0.0
15	0.0	0.0	0.0	0.0	0.0	1.0	0.0	0.0	1.5	0.7	00	0.0
16	0.0	0.0	0.0	0.0	0.0	0.0	0.0	0.0	0.0	0.0	1.8	0.0
17	0.0	0.0	0.0	7.2	0.3	0.0	0.0	0.0	0.0	6.6	5.5	3.2
18	0.0	2.0	0.0	0.0	0.0	0.5	0.0	0.0	0.0	11.0	11.2	1.2
19	0.0	0.0	0.0	0.0	0.0	0.0	4.5	0.0	0.0	00	1.0	0.0
20	0.2	0.0	0.0	0.0	13.8	0.0	0.0	0.0	1.5	0.2	6.5	0.0
21	0.0	0.0	0.0	0.0	0.0	0.0	0.0	0.0	0.0	0.0	0.0	9.5
22	0.0	0.0	0.0	0.0	0.0	7.2	12.4	5.0	0.0	1.2	4.5	0.2
23	0.0	1.1	0.0	0.0	0.0	1.8	9.5	0.0	0.0	0.0	0.0	0.0
24	5.6	0.0	0.0	0.0	0.0	0.0	0.0	0.0	0.0	0.0	0.0	0.0
25	0.0	0.0	0.0	0.0	0.0	0.0	12.5	5.0	0.0	1.0	0.0	3.0
26	0.0	0.0	1.8	0.0	0.0	0.2	0.0	0.0	0.0	0.0	0.0	27.5
27	0.0	0.0	5.8	0.0	0.0	0.0	0.0	0.0	0.0	0.0	0.0	4.5
28	1.8	0.0	0.0	4.4	0.0	22.2	1.0	1.0	0.4	0.0	0.0	0.0
29	0.0	-	6.4	0.0	0.5	0.0	0.0	0.0	0.0	0.0	3.5	0.0
30	0.0	-	0.0	0.0	0.0	0.0	0.0	0.0	0.0	0.0	2.8	0.0
31	0.0	-	12.8	-	0.0	-	0.0	0.0	-	0.0	-	0.0

**Monthly average**	28.5	8.8	46.0	23.4	25.4	43.0	71.0	13.0	51.2	20.8	80.8	80.4

Table S4. The effect of manure application (M), mineral (Min) fertilization, and M x Min interactions on the content of LMW PAHs, HMW PAHs, and the total content of 16 PAHs in soil.

**Treatment**	**$N_0P_0K_0$**	**$N_1P_1K_1$**	**$N_2P_1K_1$**	**$N_3P_1K_1$**	**$N_2P_1K_2$**	**$N_2P_1K_3$**	**$N_2P_1K_2Mg$**	**$N_2P_1K_2MgCa$**	**Mean**
					**LMW PAHs**				
Manure - µg kg^{-1}	232.9	254.3	313.0	326.0	245.0	271.3	241.0	330.2	277.3
(log transformed data)	(2.245^{g})	(2.337^{de})	(2.462$^{ab)}$	(2.453^{b})	(2.284^{f})	(2.382^{c})	(2.317^{e})	(2.477^{a})	(2.370)
Without manure - µg kg^{-1}	165.6	232,3	243.4	223.2	227.4	201.5	184.3	199.7	209.7
(log transformed data)	(2.158^{i})	(2,347^{d})	(2.361^{cd})	(2.282^{f})	(2.323^{e})	(2.193^{h})	(2.223^{g})	(2.236^{g})	(2.265)
Mean - µg kg^{-1}	199.2	243.3	278.2	274.6	238.7	236.4	212.6	265.0	-
(log transformed data)	(2.201^{g})	(2.342^{c})	(2.412^{a})	(2.368^{b})	(2.304^{d})	(2.287^{e})	(2.270^{f})	(2.357^{b})	-
					**HMW PAHs**				
Manure - µg kg^{-1}	114.7	92.3	76.8	124.0	75.1	90.9	101.3	158.4	104.2
(log transformed data)	(2.005^{cd})	(1.882^{f})	(1.737^{h})	(2.008^{cd})	(1.811^{g})	(1.936^{e})	(2.002^{cd})	(2.191^{a})	(1.946)
Without manure - µg kg^{-1}	105.8	79.0	108.5	127.3	150.2	89.7	124.1	111.2	112.0
(log transformed data	(1.982^{d})	(1.868^{f})	(2.027^{c})	(2.058^{bc})	(2.164^{a})	(1.938^{e})	(2.063^{b})	(2.003^{cd})	(2.013)
Mean - µg kg^{-1}	110.2	85.7	92.6	125.6	112.6	90.3	112.7	134.8	-
(log transformed data)	(1.993^{c})	(1.875^{e})	(1.882^{e})	(2.033^{b})	(1.988^{c})	(1.937^{d})	(2.032^{b})	(2.097^{a})	-
					**Total (16) PAHs**				
Manure - µg kg^{-1}	347.5	346.6	389.8	445.0	325.1	362.2	342.3	488.6	381.5
(log transformed data)	(2.502^{d})	(2.486^{de})	(2.562^{c})	(2.601^{b})	(2.472^{de})	(2.530^{cd})	(2.504^{d})	(2.671^{a})	(2.541)
Without manure - µg kg^{-1}	271.4	311.3	351.9	350.5	377.6	291.2	308.4	310.9	321.6

(log transformed data	(2.402^{f})	(2.479^{de})	(2.530^{cd})	(2.487^{de})	(2.571^{bc})	(2.403^{f})	(2.456^{e})	(2.456^{e})	(2.473)
Mean - µg kg^{-1}	309.4	329.0	370.9	400.2	351.3	326.7	325.3	399.7	-
(log transformed data)	(2.452^{d})	(2.483^{c})	(2.546^{a})	(2.544^{a})	(2.521^{b})	(2.467^{cd})	(2.480^{c})	(2.563^{a})	-

log transformed data marked with the letters a, b, c, d, e, f, g differ significantly at $p < 0.05$

Table S5. The effect of sampling date (D) and the interactions between sampling date (D) and manure (M) fertilization on the content of LMW PAHs, HMW PAHs, and the total content of 16 PAHs in soil.

**Date**	**April**	**May**	**August**	**September**	**Mean**
			**LMW PAHs**		
Manure - µg kg^{-1}	318.9	444.8	145.2	200.4	277.3
(log transformed data)	(2.429^{c})	(2.640^{a})	(2.140^{f})	(2.270^{e})	(2.370)
Without manure - µg kg^{-1}	200.1	324.6	94.5	219.6	209.7
(log transformed data)	(2.299^{d})	(2.504^{b})	(1.950^{g})	(2.308^{d})	(2.265)
Mean - µg kg^{-1}	259.5	384.7	119.8	210.0	-
(log transformed data)	(2.364^{b})	(2.572^{a})	(2.045^{d})	(2.289^{c})	-
			**HMW PAHs**		
Manure - µg kg^{-1}	81.5	93.6	66.2	175.5	104.2
(log transformed data)	(1.837^{g})	(1.958^{e})	(1.766^{h})	(2.225^{a})	(1.946)
Without manure - µg kg^{-1}	117.0	106.2	83.7	141.1	112.0
(log transformed data)	(2.053^{c})	(1.996^{d})	(1.885^{f})	(2.118^{b})	(2.013)
Mean - µg kg^{-1}	99.2	99.9	75.0	158.3	-
(log transformed data)	(1.945^{c})	(1.977^{b})	(1.826^{d})	(2.171^{a})	-
			**Total (16) PAHs**		
Manure - µg kg^{-1}	400.4	538.4	211.4	375.9	381.5

(log transformed data)	(2.571^{c})	(2.724^{a})	(2.311^{f})	(2.557^{c})	(2.541)
Without manure - μg kg^{-1}<br>(log transformed data)	317.0<br>(2.498^{e})	430.7<br>(2.627^{b})	178.1<br>(2.229^{g})	360.7<br>(2.537^{d})	321.6<br>(2.473)
Mean - μg kg^{-1}<br>(log transformed data)	358.7<br>(2.535^{c})	484.6<br>(2.676^{a})	194.8<br>(2.270^{d})	368.3<br>(2.547^{b})	-<br>-

log transformed data marked with the letters a, b, c, d, e, f, g differ significantly at $p < 0.05$

Table S6. Factor loadings in April, May, August, and September.

**Variable**	**April**				**May**				**August**				**September**			
	PC1	PC2	PC3	PC4	PC1	PC2	PC3	PC4	PC1	PC2	PC3	PC4	PC1	PC2	PC3	PC4
Organotrophic bacteria	**0.85**	-0.47	-0.04	-0.05	-0.28	**-0.74**	-0.03	-0.29	**-0.75**	-0.46	0.19	0.05	**-0.72**	-0.32	0.01	0.13
Ammonifying bacteria	**0.71**	-0.47	-0.07	-0.39	-0.49	0.12	**-0.70**	-0.06	0.15	-0.45	-0.22	0.38	**-0.67**	0.12	0.17	0.55
Nitrogen-fixing bacteria	**0.69**	-0.47	-0.19	-0.35	-0.49	0.17	**-0.71**	-0.24	-0.57	**-0.63**	0.17	0.21	**-0.87**	-0.14	0.07	0.22
Actinobacteria	**0.79**	-0.53	0.09	-0.11	**-0.63**	-0.02	**-0.65**	0.20	**-0.61**	-040	0.41	-0.32	**-0.83**	-0.12	0.24	0.17
Fungi	0.57	-0.41	-0.34	-0.23	-0.37	-0.15	-0.51	**0.66**	-0.53	-0.54	002	-0.37	-0.36	-0.44	**0.67**	-0.19
Dehydrogenases	**0.60**	0.45	**0.60**	-0.09	**-0.85**	0.27	0.36	0.09	**-0.74**	0.45	-0.29	0.19	**-0.84**	0.19	-0.21	-0.27
Catalase	**0.90**	0.20	-0.19	0.24	**-0.83**	016	0.15	0.24	-015	0.06	0.43	-0.22	**0.60**	0.04	-0.28	-0.48
Urease	**0.80**	0.18	0.14	-0.19	**-0.64**	0.21	-0.13	-0.39	**-0.90**	0.09	-0.06	0.18	**-091**	0.23	-0.05	-0.22
Acid phosphatase	0.43	**0.61**	-0.48	-0.13	**-0.77**	-0.35	0.20	0.35	**-0.81**	0.31	-0.04	-0.40	**-0.72**	0.22	0.35	-0.36
Alkaline phosphatase	**0.73**	-0.42	0.27	0.21	**-0.85**	0.36	0.11	-0.18	**-0.88**	0.03	0.33	0.08	**-0.88**	0.01	-0.34	-0.04
C	**0.79**	0.54	-0.06	-0.17	**-0.85**	-041	0.13	0.06	**-0.77**	0.40	-0.13	-0.33	**-0.92**	0.08	0.13	-0.23
N	**0.76**	0.32	-0.36	0.05	**-0.73**	-0.37	0.30	0.22	**-0.69**	0.35	0.19	-0.17	**-0.82**	0.26	0.20	-0.26
Hh	**0.60**	-0.14	**-0.71**	0.03	**0.67**	**-0.61**	-0.06	0.23	**0.72**	0.05	-0.05	**-0.62**	**0.71**	0.05	**0.65**	-0.14
pH	**0.69**	0.38	0.57	-0.02	**-0.81**	049	0.12	-0.16	**-0.91**	0.09	0.03	0.34	**-0.83**	0.08	-0.25	0.15
LMW PAHs	**0.74**	0.16	-0.35	0.49	-0.49	**-0.75**	0.19	-0.24	-0.47	0.11	**-0.82**	-0.10	0.06	**-0.88**	0.10	0.03
HMW PAHs	0.07	**-0.60**	0.36	0.53	0.23	-0.41	-0.58	-0.14	-001	**-0.87**	-0.13	-0.16	-0.35	**-0.69**	-0.34	-0.24
Total (16) PAHs	**0.70**	-0.04	-0.21	**0.67**	-0.39	**-0.84**	-0.01	-0.27	-0.38	-0.48	**-0.73**	-0.19	-0.13	**-0.97**	-0.10	-0.09
Eigenvalues	8.22	2.84	2.15	1.52	7.04	3.38	2.41	1.27	7.16	2.88	1.95	1.43	8.58	2.75	1.59	1.15
% explained variance	**48.3%**	**16.7%**	**12,7%**	**8.9%**	**41.4%**	**19.9%**	**14.2%**	**7.4%**	**42.1%**	**17.0%**	**11.5%**	**8.3%**	**50.5%**	**16.2%**	**9.3%**	**6.7%**
